# Shedding Light onto the City Blues Myth—The Potential of Stimulating and Activating Effects of Urban Public Spaces and the Role of City Relatedness

**DOI:** 10.3390/ijerph19137606

**Published:** 2022-06-21

**Authors:** Lena Lämmle, Eike von Lindern, Dorothee Rummel, Mark Michaeli, Matthias Ziegler

**Affiliations:** 1 Medical School Hamburg, University of Applied Sciences and Medical University, 20457 Hamburg, Germany; 2 Dialog N—Research and Communication for People, Environment and Nature, 8006 Zurich, Switzerland; eike.von.lindern@dialog-n.ch; 3 TUM School of Engineering and Design, Technical University of Munich, 80333 Munich, Germany; dorothee.rummel@tum.de (D.R.); michaeli@tum.de (M.M.); 4 Faculty of Life Sciences, Humboldt-Universität zu Berlin, 10117 Berlin, Germany; zieglema@hu-berlin.de

**Keywords:** perceived restorativeness, nature relatedness, city relatedness

## Abstract

The present study aims to investigate whether a sense of relatedness to a city helps to broaden understanding of the restorative potential of urban public spaces. Findings based on a sample of German adults (*n* = 249) confirm that people experience relatedness to a city. The study’s 3 × 3 (built, mixed, natural environment) × (average, livability environment, bird’s-eye view) design revealed disordinal interactions for being away, fascination, preference, mental fatigue, and stimulating and activating effects associated with cities. This implies that humans’ place perceptions are more complex than previously assumed. Both city and nature relatedness were relevant covariates of these findings. Surprisingly, the construct ‘activating effects’, was found to be mostly perceived as more positive for mixed and built environments compared to natural environments. Thus, complementing restorative environments research by introducing a measure for city relatedness significantly enhances understanding of the potential of urban public spaces for promoting human health and well-being.

## 1. Introduction

It is expected that in 2050 more than 68 percent of the world’s population will live in cities [1]. According to the World Health Organization, unsustainable and unplanned urban development poses health risks for current and future urban dwellers. Among the risks mentioned are diminishing public urban green spaces, such as parks and gardens. One reason why this is considered a risk is that those green sites provide opportunities for physical activity, social interaction, and recreation [2]. Recreation, as a fundamental psychological need [3], can be traced back to the biophilia hypothesis [4]. It states that human beings are genetically predisposed to respond positively to natural environments, as this is the environment in which humanity evolved. Accordingly, past research provides evidence for nature’s beneficial effects on cognitive and affective functioning, e.g., [5,6,7,8], as well as on quality of life, subjective health, and heart rate [9]. However, depending on the spatial design, urban sites such as pedestrian areas or built environments in historic styles also have restorative potential [8,10] and can be associated with lower physiological parameters of cortisol concentration [7] or increased heart rate variability [11]. The latter two studies compared green environments to urban environments with high architectural quality and consistently found that none of the physiological parameters considered exhibited significant differences between green and urban environments; both environment types can be framed as restorative environments. However, both studies confirm previous findings of a more complete perceived restorativeness for green compared to urban environments [7,11] (This difference in perceived restorativeness and physiological markers should be interpreted carefully, as past stress research has delivered inconsistent findings for measures of perceived stress and biological stress (e.g., [12,13]. The same is true for the long-held assumption that perceived and physical restorativeness are correlated [14]). On the other hand, like with urban grey settings (e.g., heavy motor traffic density; [8]), there is also evidence for unpleasant natural settings that tend to increase levels of stress and attention fatigue, such as low levels of prospect (constricted field of vision), high levels of refuge (places to hide; [15]), or high levels of enclosure [16]. Finally, differences in perceived restorativeness can also be observed within a given environment, independently of whether it is natural or built [17]. For example, when observing the number of green features and natural sound [18] or for blue spaces such as riversides [19]. Thus, against the background of reduced access to natural settings in an urbanizing world, combined with humanity’s fundamental psychological need for recovery from fatigue and stress, this paper responds to the call for shifting focus to investigate urban contexts that promote effective restoration and serve urban public health [3,10].

### 1.1. Perceived Restorativeness

Perceived restorativeness can be understood as the perceived degree to which depleted cognitive resources needed for directed attention over the course of a day become renewed or replenished. Whether (and to what degree) restoration is initiated was found to be strongly associated with features of the environment, e.g., [20]. According to attention restoration theory (ART), the four core elements of restorative environments are assumed to be individuals’ sense of being away from ordinary life routines; fascination, which elicits effortless attention and counters the depletion of cognitive resources; extent, as coherence between environmental features and their scope; and compatibility, which refers to the congruence between environmental qualities and people’s needs or intentions [21]. The underlying idea is that direct attention capacity diminishes over the course of the day, leading to attentional fatigue. Situations that do not require any direct attention are therefore assumed to not only allow for, but also initiate, restoration [20]. Even though completely built environments are assumed to have restorative potential, alongside natural environments, if they provide restorative properties, natural environments are assumed to always outperform built ones because information-rich cities require more mental resources to direct attention [21]. This hypothesis is supported by the finding that images of natural environments require less cognitive effort, as assessed through lower eye movement activity, than images of built environments [22]. However, another assumption derived from previous research is that complexity, which is linked to variety in stimulus pattern and the number of independently perceived elements, also correlates with perceived restorativeness [14]. 

### 1.2. Setting Attributes Linked to Restorativeness

In addition to well-designed urban environments [23], other setting information associated with restorativeness, such as livability, has also been shown to improve the restorative potential of public places. Whyte’s [24,25] seminal work identified eight elements for vibrant and inviting places, with sitting space being presented as the most important factor. Further spatial aspects that attract people are the presence of unusual elements, such as a sculpture, that can lead strangers to talk with one another and thereby support social interaction (a phenomenon known as triangulation); access to food and access to the street in order to watch people passing by; as well as deciduous trees, sunlight, and water. Although seats, triangulation, and food are considered the three key elements [26], findings suggest that the presence of only two of these three key elements improves restorativeness more than all three key elements together [27]. An analysis of urban green spaces identified eight relevant perceived sensory dimensions (PSDs), which bear some similarities to Whyte’s livability theory [24,25]: culture (such as sculptural elements), social (such as food/restaurant, entertainment), refuge (such as sitting areas, play equipment), nature (e.g., untouched/wild), space (e.g., free/spacious), serenity (e.g., silent/calm), richness in species (animals and plants), and prospect (e.g., flat and landscaped grass surfaces [28]). Unlike Whyte’s livability theory [24,25], which emphasizes the presence of two to three key elements, preference for environment was initially shown to increase with the number of PSDs [29]. However, in a later study on PSDs, the strongest perceived restorativeness was not observed for places equipped with many green elements, nor for places with limited green elements, but instead for places with historical buildings and access to food [28]. In this article we will use the term livability for both Whyte’s livability theory [24,25] and PSDs [29], due to their common foundation. 

#### Scale Development and Design Innovation

Choosing and describing places that either exhibit or do not exhibit livability is useful as it allows elements of a place to be categorized and practical implications to be derived [30]. However, a simple description fails to capture the underlying psychological processes that are associated with how the considered places are truly perceived. We therefore developed items to cover perceptions of the livability dimensions, e.g., whether a place is perceived as promoting social interactions or as vibrant and stimulating (see Section 2.3). In doing so, we are broadening the current item pool of how places are perceived aside from perceived restorativeness and preference [14]. This has been conducted to address the requirements for considering, (1) a greater variety of environment types, as well as (2) a greater level of detail with respect to specific environments [17]. (1) Aside from a focus on natural environments [8,18], when built environments have been considered, the focus was either on the livability of environments, such as museum settings [31,32], or that of grey environments involving elements such as heavy motor traffic density [8]. In contrast, few studies have considered average environments [8,22] or mixed built environments that were also shown to have restorative potential [14,17,30]. Following the call for a greater variety of environment types, we have differentiated between natural, mixed, and built environments (types level); (2) and between average and livability environments, as well as the bird’s-eye view, in the present study (detailed level; see Supplementary Figure S1). We added bird’s-eye view, as living in tall buildings in cities often entails a bird’s-eye view for at least parts of the day, and should therefore be considered. To our knowledge this perspective has not been investigated before and should allow to examine whether, in addition to the type and number of a place’s elements, different perspectives also influence how we perceive places. Moreover, the bird’s-eye view might also be associated with a certain distance to road traffic and the associated consequences.

### 1.3. Human Attributes Linked to Restorativeness

The extent to which an environment is perceived as restorative depends not only on physical features of the environment, but also on individual differences such as connections to nature [3,33], as they are assumed to explain the heterogeneity of how restorative environments are perceived [34]. Past research has focused on stress-related factors such as mood, perceived stress over the past month [11], relaxation, emotions, and emotion regulation [10]. In addition, on a cognitive level, preferences for natural or urban scenes [14] and mental fatigue [35] have been shown to be related to the perceived restorativeness of environments. Moreover, people who do not perceive nature as restorative do not develop a preference for natural environments [36]. Conversely, people who have a connection to nature also perceive it as more restorative [37]. Connectedness to nature is defined as an affective and experiential connection to natural environments [38]. The experiential connection seems to be increasable at any life stage, independently of whether a person lives in a city [39].

#### Scale Development

As humanity already has years of experience living in (or with) cities, it is reasonable to assume that humans have also developed a connectedness to the city. Cities provide certain amenities, such as opportunities for social interaction and cultural engagement, and health care facilities. Therefore, drawing upon theory and research on connectedness to nature, we developed a measure to assess city relatedness (see Section 2). In line with the documented relation between the perceived restorativeness of natural environments and connectedness to nature [37], we expect to find a similar association between perceived restorativeness of (mixed) built environments and city connectedness. When such associations appear, they might influence how the considered (mixed) built environments are perceived in terms of livability and activating effects. 

## 2. Materials and Methods

### 2.1. Sample, Informed Consent and Ethical Principles

A total of 249 adults (70.7% female) with a mean age of 26.83 years (*SD* = 11.62, range = 18–76 years) participated in the online study. This sample size is close to Schönbrodt and Perugini’s [40] recommended sample size of 260 participants for a 90% confidence level and a confidence interval width of 0.10 (as the smallest corridor of stability around the true value), or the more general recommendation of 250 participants for typical scenarios. Complete data were available for all 249 participants. Informed consent was obtained from the participants online. They were informed that their participation is anonymous, completely voluntary, and that they can terminate their participation in the study at any time without any negative consequences. Participants were also informed that the collected information would only be used for the present study. Subjects were thanked and debriefed after participation. The current study was conducted in Germany. Standard ethical approvals are not standard in Germany for this study type. Ethical approval is typically requested for grant proposals or clinical studies. This study received no funding. While planning the study, great care was taken to make sure that the study protocol adhered to the APA’s Ethical Principles of Psychologists and Code of Conduct. 

### 2.2. Study Design 

Photographs were used as stimuli, as they have frequently been used in past research [10]. Moreover, exposure to such photographs has been shown to improve emotional states and cognitive functioning (e.g., [41]). The photographs were taken with a common smartphone camera to obtain a classic tourist shot and impression of the sites considered. The photos were taken during the week of December 6 to 13, between 12:00 and 2 p.m. on sunny days in the center of Munich. Munich is considered one of the least stressful cities worldwide (Rank 5; [42]) and has more than 1.5 million inhabitants, making it the third-largest city in Germany [43]. However, as Ratcliff and Korpela [44] point out, memories of a place enhance place identity and thus perceived restorativeness. Therefore, we decided to control for this variable by asking students from a university in Hamburg and members of their social network to participate in the present study. Hamburg is the second largest city in Germany, with more than 1.8 million inhabitants [42] and the ninth least stressful city worldwide [42]. Descriptive data revealed that most of the participants lived in the city of Hamburg or nearby; 16 (6.4%) of them reported having previously lived in Munich. We further asked whether or not participants recognized each of the depicted places. A total of 100 participants (40.2%) indicated familiarity with at least one of the nine places. 

For categorization and to derive practical implications, each place was classified according to livability/PSDs and their relevant cues (see Supplementary Table S1; following Peschardt and Stigsdotter [30]). However, we made some changes to include further livability aspects [24,25], as we did not only consider small public urban green spaces. Moreover, some of the cues were listed as markers for several sensory dimensions, leading to overlap among those dimensions. Hence, the second aim of our revisions was to develop more precisely defined dimensions. By deleting repetitions and reassigning some cues that seemed to fit to another dimension (e.g., the cue ‘places where people can gather’ was assigned to the social rather than the space dimension; the cue ‘a lot of trees’ was assigned to the nature rather than the space dimension; the cue ‘exhibitions’ was assigned to the culture rather than the social dimension). Furthermore, we deleted cues irrelevant for the present study such as ‘several species of animals or foreign plants’. We then broadened the dimension ‘rich in species’ to ‘rich in information’, in order to incorporate previous research [22] and better align with the places considered. Finally, we redefined the cues for refuge, in order to bring them more in line with ART [21,45]. A group of experts rated the PSD and agreed upon the consensus rating used here. 

Participants were first asked to answer questions regarding their socio-demographics and whether they enjoy nature trips (*M* ± *SD* = 4.31 ± 0.82) over city trips (*M* ± *SD* = 4.04 ± 0.99; *t*(248) = −3.53, 2-tailed, *p* < 0.05, *d* = 0.30; 1 − ß > 0.80). They then filled out the Nature Relatedness Scale NRS [46] and the City Relatedness Scale (CRS; see below) in randomized item order. Subsequently, the three places from the types condition (built, mixed, natural), as well as the detailed condition (average, livability, mixed), were presented in random order and participants were asked to rank them (see Supplementary Table S2). The inverse randomization process (random order for the types condition followed by random order for the detailed condition) was used to ask participants to rate their perceived restorativeness, preference, mental fatigue, and the newly developed items (see below) for each place. 

### 2.3. Measures

#### 2.3.1. Being Away and Fascination

As fascination [47] and being away [35] are assumed to play a crucial role in restoration according to ART, we focused on these two of the four ART facets operationalized in the PRS by [48]. Different scales have been used in previous studies (e.g., 7-point rating scale, [44]; 11-point scale, e.g., [30]) as answering options for the Perceived Restorativeness Scale (PRS; 0 = not at all; 6, 10 = completely). In our study, given that participants first rated the NRS and CRS, both of which use a five-point rating scale ranging from 0 = strongly disagree to 4 = strongly agree, we remained with these response options throughout the entire questionnaire, yielding Cronbach’s *α* values between 0.84 and 0.90 (see Table 1), which is in line with past findings (e.g., [49]). 

#### 2.3.2. Nature Relatedness and City Relatedness

Connectedness or relatedness to nature has been operationalized using different scales. The most systematically studied measures are the Connectedness to Nature Scale [38] and the NRS [46]. The NRS seemed most appropriate for developing a comparable measure to assess connectedness or relatedness to cities. Unlike the CRS, the NRS captures experiences with nature, which also seems quite relevant for city relatedness. The NRS comprises an overall nature relatedness factor (Cronbach’s α = 0.87), as well as the three dimensions of self (i.e., how strongly is one’s identification with the natural environment, α = 0.84), perspective (i.e., the extent to which one’s relationship with the natural environment is manifested in their attitudes and behavior, α = 0.66), and experience (i.e., physical familiarity with and attraction to the natural environment, α = 0.80; [46]). However, as the NRS short form did not include an item for the perspective facet [50], and as these items and thus also the facet itself are not transferable to city relatedness, we decided to focus on the experience and self-facets. Reliabilities for the present study are reported in Supplementary Table S3. In developing the CRS, we aimed to mirror the NRS items as closely as possible (e.g., NRS: ‘I take notice of wildlife wherever I am’; CRS: ‘I take notice of wildlife wherever I am’). When this was not possible, we focused on transferring the main idea of the item and facet (e.g., NRS: ‘I enjoy digging in the earth and getting dirt on my hands’; CRS: ‘I like to make use of the diverse offers of the city’). All items can be found in Table S4 of the Supplemental Material. Reliability estimates for the CRS were very similar to those for the NRS in the present data (see Supplementary Table S3). 

#### 2.3.3. Preference and Mental Fatigue

Preference was assessed with a commonly used single item (‘I like the place’; e.g., [17,30]). To assess the degree of mental fatigue, we slightly modified one of the two items by von Lindern [35] for assessing the object aspect of setting characteristics. Instead of ‘during leisure time’, we introduced the item with the words ‘at this place’. The item thus reads: ‘At this place, specific objects remind me of mental demands or fatigue just through their presence’. Afterwards, participants were asked to provide details about these objects. We used the same answer options as for ‘being away’ and ‘fascination’ for both the preference and mental fatigue items. The responses to the question asking participants to name objects whose mere presence reminded them of mental demands or fatigue are listed in Supplementary Table S8.

#### 2.3.4. Newly Developed Setting-Related Items

To assess further characteristics of natural and urban places besides perceived restorativeness, preference, and mental fatigue, we referred back to the six dimensions of place memories: activities within the place, cognitive and emotional responses, social context, environment, time, and self (note that we excluded the ‘activities within the place’ dimension because of its descriptive character [44]); and to the urban happiness factors of appearance, functional possibilities, atmosphere, and social life [51,52]; as well as to the CRS. Because of the proximity to Ratcliff and Korpela’s [44] dimensions of cognitive and emotional responses and self, we further examined the motivational reasons for interior design in terms of identitying claims directed towards the self and others, as well as to regulation of one’s thoughts and feelings [53]. 

We thought activities within the place and functional possibilities share a common underlying idea and therefore formulated the corresponding item as follows: ‘This place provides a broad scope for action’. Likewise, social aspects are assumed to play a role in both theories (as well as others mentioned above). We thus formulated the corresponding item as follows: ‘This place encourages social life’. As both of these items share a common idea of what a place can offer, we added two further items in this direction, ‘This place provides amenities’ and ‘At this place, you are at the cutting edge’, followed by the time aspect and the two CRS items ‘Even in the middle of nature, I think about the amenities in the city’ and ‘My ideal vacation spot would be a vibrant metropolis’. In line with this vibrancy aspect, as well as the emotional response of vitality to a known place, we added the following item: ‘This place has something invigorating’. Moreover, we formulated one item for each motivational reason for interior design, as well as the self, cognitive, and emotional response dimensions as follows: ‘In this place, I recognize myself’ (identity claim to self), ‘I would meet my friends at this place, so that they can learn more about me’, (identity claim to others), ‘This place regulates my emotions in a positive sense’ (emotion regulation), and ‘This place regulates my thoughts in a positive sense’ (thought regulation). As the aesthetic aspect of a place is part of the environment dimension and a happiness factor, we formulated a corresponding item as follows: ‘This place creates a pleasant atmosphere’. With regard to the appearance aspect, the corresponding item was: ‘This place has a stimulating appearance’. Besides appearance, the characteristics of a place also play an important role. We therefore added the item: ‘This place has stimulating characteristics’. Finally, addressing the recreational aspect, we formulated the item: ‘This place invites relaxation’ (for an overview, see Supplementary Table S5). Because the underlying theories used for item formulation partly overlap, we did not hypothesize a specific number of factors for these items.

### 2.4. Statistical Analyses

Statistical analyses were performed using R [54] and R Studio [55]. Structural equation modelling (SEM) using the lavaan package [56] was used to verify the structure of nature and city relatedness measures. To assess model fit, the χ^2^-test, comparative fit index (CFI), root mean squared error of approximation (RMSEA), and standardized root mean squared residual (SRMR) were considered. Hu and Bentler’s recommendation that a good model fit is reflected by a CFI close to 0.95, a SRMR smaller than 0.08 and a RMSEA smaller than 0.06 should be seen as desirable values rather than strict cut-off criteria [57]. To determine the number of factors for the newly developed setting-related items, the minimum average partial (MAP) test, Horn’s parallel analysis, and eigenvalues were considered before conducting an exploratory factor analysis (EFA). To assess the feasibility of the data, we considered the Kaiser-Meyer-Olkin coefficient and the Bartlett test. All of these analyses were conducted with the psych package [58]. To evaluate the 3 × 3 design (built, mixed, nature × average, livability, bird’s-eye perspective), and in order to test whether nature and city relatedness moderate the effects of these conditions, we ran a series of ANCOVAs, in which each of the constructs acted as a covariate. The tidyr package was used for the ANCOVAs [59]. Tukey method was used for post hoc comparisons.

## 3. Results

### 3.1. Human Attributes: Nature and City Relatedness 

In order to be able to classify the results from the newly developed CRS, we conducted the same analyses as for the NRS. The SEM for nature-related self and nature-related experience revealed unsatisfactory model fits (see Supplementary Table S6 for SEM findings). Modification indices suggested that the items ‘I always think about how my actions can affect the environment’ and ‘I am very aware of environmental issues’ shared common variance. After adding this residual correlation (*r* = 0.39), the model fits were acceptable. The correlation between nature-related self and nature-related experience amounted to *r* = 0.87. Because of the small difference in effect, we decided to consider nature relatedness in the subsequent analyses. Acceptable model fits were also observed for city-related self and city-related experience. The correlation between these facets amounted to *r* = 0.85. The correlations between nature and city were close to zero on both the domain and facet level. Moderate differences were observed between nature and city relatedness and self, with higher means and thus manifestation for nature. No difference was observed when comparing nature and city experience (see Supplementary Table S3). 

### 3.2. Setting Attributes: Perceived Restorativeness, Stimulating and Activating Effects, Preference, and Mental Fatigue

With regard to the places’ preferences, participant’s first preference in the average condition was the nature condition, followed by the mixed and built conditions. In the livability condition, the mixed environment was preferred over the nature and built conditions. The difference between the nature and the mixed conditions for the bird’s-eye perspective was 0.8%, and both were preferred over the built condition (see Supplementary Table S2). 

The Bartlett test and KMO coefficients indicated that the presented data are appropriate for EFA (see Supplementary Table S5). For the nine places, the MAP test, Horn’s parallel analysis, and eigenvalues largely suggested a two-factor solution for the 13 items; only Horn’s parallel analysis proposed a three-factor solution in three of nine cases. We therefore considered both factor solutions, but the findings for the two-factor solutions depicted in the table were repeatedly found to be more stable. The only item that shifted between the two factors to an almost equal extent is ‘This place has something invigorating’. However, as it loaded once more onto the second factor, this item was assigned to the second factor. As the first factor encompasses items referring to stimulating effects, we labeled the factor accordingly. As the second factor encompasses items referring to activating effects, we again labeled the factor accordingly. Reliability estimates were satisfactory and are depicted in Table 1.

Overall, the findings of the ANOVAs and ANCOVAs (Significance level did not differ between ANOVAs and ANCOVAs. Effect sizes only differed in some cases and then to a small extent (third decimal place). We therefore decided to report the ANCOVA values, as at least one of the covariates was significant for each construct considered. ɳ^2^ differed in two cases when nature and city relatedness were both significant. Again, as the extent of the difference was small (third decimal place), we always depicted the smaller value. The *p*-value only differed for mental fatigue in the detailed condition, but in both cases the finding was not significant) consistently revealed small to moderate significant main effects for the types condition (built, mixed, nature), small significant main effects for the detailed condition (average, livability, bird’s-eye perspective), and small significant effects for the interaction terms for all of the considered scales except for mental fatigue, where the detailed condition was not significant (see Table 1).

Nature relatedness was a significant covariate for fascination, activating effects, and mental fatigue, but always to a very small extent. City relatedness was a significant covariate for all of the scales considered; all effect sizes were comparable to those for nature relatedness with the exception of preference, where the effect size was small. Thus, higher scores for city relatedness are associated with higher scores for preference. Interaction effects for nature and city relatedness were generally also very small, yet mostly significant. Only for activating effects in the types condition were the effect sizes small and significant.

Post-hoc comparisons for the types condition were significant for all scales considered (except for mental fatigue in the mixed-nature comparison). While the means for being away, fascination, stimulating effect, preference, and mental fatigue were higher (lower for mental fatigue) in the nature condition, followed by the mixed and built conditions, it was exactly the opposite for activating effects. Moreover, activating effects were positively associated with both restorative facets. The same applies for stimulating effects and preference, while mental fatigue seems to be unrelated to these variables (see Supplementary Table S7).

The post-hoc comparisons for the detailed condition revealed significant differences between the average and livability conditions and between the average and bird’s-eye perspective conditions for all scales considered except for mental fatigue, where none of the post-hoc tests were significant. The comparison between the livability and bird’s-eye view conditions was only significant for fascination. As can be seen in Supplementary Figure S2 (also see Section 4), all of the interactions were disordinal, meaning that the interaction effects can be interpreted, but the principal effects cannot. Confidence intervals show that most of the pairwise comparisons are significant.

## 4. Discussion

The present study provides several new insights into people’s relationship to natural, mixed, and built environments. First, it could be shown that people can experience relatedness not only to nature [46], but also to a city. Furthermore, both nature and city relatedness are associated with how we perceive a place, therefore supporting the assumption that individual differences moderate our perceptions [34]. Second, the present findings support previous findings that mixed and natural environments can be preferred to an equal extent [14]. Indeed, in the present study, the mixed environment even outperformed the natural environment in the livability condition with regard to perceived restorativeness. Reasons for this might be, on the one hand, that in the mixed condition the meadow is greener and the colors are more intense overall, since colors are relevant for the characterization of vegetation [60]. On the other hand, the composition [59] of the city park characteristic versus the freer growth (PSD; Table S1) may be another reason. Finally, qualitative questioning on addressed cues suggests the crowd and concrete sidewalk in the natural condition (Table S8). Third, the bird’s-eye perspective was shown to have comparable potential for perceived restorativeness as livability environments [24,25,28], and both have more potential than an average environment, therefore emphasizing the need to consider this perspective in the future. Fourth, the 3 × 3 design of types (built, mixed, nature) and detailed (average, livability, bird’s-eye perspective) setting characteristics revealed that these are not independent, implying that humans’ place perceptions are more complex than previously assumed. Hence, the present findings only partly confirm previous findings of higher perceived restorativeness and preference [10] and less mental fatigue [35] in natural environments, followed by mixed and built environments. Our disordinal interactions yielded the following results: in four cases, the built environment outperformed the mixed environment (average condition for fascination, stimulating and activating effects, and preference), for which the reasoning might be the more public setting in the built environment condition, because of the shop and the restaurant with sitting options outside, as well as the wide view and the greater variety of houses (PSD; Table S1). In seven cases, the mixed environment outperformed the natural environment (livability and bird’s-eye perspective condition for fascination and activating effects; livability condition for stimulating effects, preference, and less mental fatigue). Here, the mixed bird’s-eye view includes historic buildings (also recognized as cues from participants; see Table S8), an artist-designed treetop perspective, a sculpture, and seating. In addition to the above information, the mixed livability condition also offers seating (PSD; Table S1). In one case, the built environment outperformed the natural environment (bird’s-eye perspective condition for activating effects). This might be due to the information richness of the cues, the wide view with different characteristics, possibilities for different activities, and locomotion options (PSD; Table S1). Fifth, two new theoretically based constructs for the evaluation of environmental characteristics were successfully operationalized, namely the stimulating and activating effects of a place, indicating that our places’ perceptions are more diverse than previously assumed. Sixth, as noted above, ‘activating effects’ were in some cases perceived as higher for the built environment compared to the mixed environment, while both were perceived as higher compared to the natural environment (see above). Moreover, ‘activating effects’ were positively related to perceived restorativeness, lending further support to the urban happiness approach [51]. Stimulating effects were also strongly associated with being away and fascination. Seventh, apart from these new constructs, the present study’s qualitative findings support the necessity of further exploring characteristics of environments (in accordance with findings by Ratcliff and Korpela [44]). Eighth, the qualitative findings point to several new PSD cues [28], especially with respect to a possible new urban dimension (e.g., facades, concrete). They also suggest a need to consider more differentiated cues. For example, the social aspect was considered positive in previous studies, but many participants in the present study perceived it as rather negative.

The present study also has some limitations that must be considered. Although participants lived in a different city than the city where the photographs were taken, one hundred of them indicated being familiar with at least one place used in the study. However, in the open answer format for the mental fatigue item, some participants wrote the name of the place or the area where they thought the picture had been taken. None of the participants were right, as they all thought the pictures had been taken in Hamburg. Nevertheless, those participants stated that they recognized the place. Hence, future research should employ an open response format for each place, in order to verify whether participants are actually familiar with the place and to control for socially desirable responses, as some participants might have had the impression that they should recognize the place. Moreover, participants should be asked whether they have ever been to this place and if so, whether they have place memories, in order to appropriately control for such place memories [44]. The participants in this study might also have recognized the depicted places from pictures they had seen. Even though we controlled for whether participants have ever lived in Munich, we decided to not exclude these participants, as they indicated that they only recognized some of the places, just like the participants who had never lived in Munich. Nevertheless, we also analyzed AN(C)OVAs excluding participants who indicated recognizing at least one place and excluding participants who have ever lived in Munich (but did not necessarily recognize any of the places). Some of the findings differed in these analyses, suggesting moderation effects. However, as we cannot verify whether participants actually recognized the correct place or have corresponding place memories, further research incorporating these aspects is strongly recommended. Likewise, when comparing (previous) inhabitants and non-inhabitants, a sample size larger than n = 16 is needed to obtain stable results. Based on the present qualitative findings, we also suggest asking about not only negative but also positive place cues, in order to enrich the existing pool of cues and PSDs [28]. Regarding livability PSDs, in addition to descriptive cues, future studies should address in more detail cues derived from qualitative data reflecting how participants perceive a place, such as togetherness [44], safety [61], arousal, liveliness, curiosity, and exploration [62]. Finally, as water is assumed to contribute to livability [19], the three places chosen for the livability condition should include water elements, which was the case in this study. However, as the pictures were taken in December, the fountains in the built and mixed condition were covered. This might have had a buffering or enhancing (mental fatigue) effect on how participants perceived these places compared to the nature condition. Likewise, the average natural place included water elements, but the other average places did not; they instead included seating areas, so as to try to balance out livability elements [27]. Another limitation of winter surveys is that we cannot apply the nature-relevant classifications of diversity, species richness, and composition [63], even though the results on diversity of vegetation with respect to preference are mixed [60]. Nevertheless, these classifications are important for studying seasonal differences in the perception of built, mixed, and natural urban settings.

These limitations notwithstanding, the present findings have implications for theory and practice. Our initial findings confirm the city relatedness construct and call for future research to determine the factors that affect it. Moreover, as city relatedness was shown to be associated with how we perceive an environment, an increase in city relatedness probably also leads to increased positive perceptions of urban environments. Finally, these findings imply a change in approach to urban happiness, as the location-specific focus would shift to a more interactionist perspective. An examination of further individual differences would shed light onto different perceptions of a place [34,62] and might help to explain the divergent findings, such as that togetherness seems to be relevant for place memories [44], despite ‘a lot of people’ being mentioned as a cue associated with mental fatigue in the present study. Moreover, as social interactions are part of urban life, a single item such as the one used in the present study might not fully capture their complexity. This could be enriched through a broader and deeper understanding rooted in a social psychology perspective. Hence, in addition to an interactionist approach, a more interdisciplinary approach is needed to understand which human and environmental factors affect our environmental perceptions. The present study was just the first step in this direction. We integrated several theories and approaches from different disciplines to examine the potential for elements other than restorativeness in environments, using a single item approach. For example, like the social aspect, identity claims and vitality have been shown to affect our environmental perceptions; but again, future research should investigate whether these constructs are broader than initially assessed in the present study. Thus, our conclusion is not that stimulating and activating effects must be considered as new constructs alongside restorativeness. Rather, our conclusion is that these constructs show that the mode of action in places is more complex than previously assumed. This conclusion is supported by the observed disordinal interaction effects of the types and detailed environmental information, which in turn support previous findings that areas enclosed by vegetation interact with the degree of urban site closure [61]. These findings further indicate an ongoing need to discriminate between different examples of the same type of setting (see also [17]). Classifications, such as PSDs [28], play a central role in their selection and the subsequent discussion of their perception by the participants. Thus, studies with different settings, but the same classification system, are needed to understand and explain the perception of built, mixed, and natural conditions. The findings of the present study contribute to the further development of the classification system used here and to the selection of specific settings based on it. Finally, we agree with Staats and colleagues’ [3] conclusion that built environments undeniably have restorative qualities.

## Figures and Tables

**Table 1:** Descriptive statistics, Cronbach’s α, and AN(C)OVAs for Being Away, Fascination, Stimulating, and Activating Effects, Preference and Mental Fatigue.

Scale		Average		Livability		Bird’s Eye View		
		*M* ± *SD*	*α*	*M* ± *SD*	*α*	*M* ± *SD*	*α*	*M* ± *SD_c_*
being away	built ^b,m,^***	2.8 ± 0.81	0.90	2.38 ± 0.75	0.87	2.21 ± 0.77	0.88	2.26 ± 0.78
	mixed ^m,n,^***	2.14 ± 0.72	0.87	3.59 ± 0.73	0.88	3.19 ± 0.77	0.88	2.97 ± 0.96
	natural ^b,n,^***	4.25 ± 0.66	0.88	3.53 ± 0.77	0.87	4.00 ± 0.70	0.87	3.93 ± 0.77
	*M* ± *SD_d_*	2.86 ± 1.23	a,l ***	3.17 ± 0.93	l,b	3.13 ± 1.05	a,b ***	
t	d	nr	cr	c*d	c*nr	d*nr	c*cr	d*cr
p|ɳ^2^ F|df								
***|0.092 2096.06|2	***|0.002 42.47|2	0.16|<0.001 2.01|1	***|0.003 132.91|1	***|0.027 299.35|4	***|0.004 80.24|2	0.58|<0.001 0.56|2	***|0.004 95.12|2	0.63|<0.001 0.46|2
		*M* ± *SD*	*α*	*M* ± *SD*	*α*	*M* ± *SD*	*α*	*M* ± *SD_c_*
fascination	built ^b,m,^***	2.62 ± 0.91	0.90	3.11 ± 0.81	0.87	3.17 ± 0.90	0.90	2.97 ± 0.91
	mixed ^m,n,^***	2.32 ± 0.81	0.89	3.66 ± 0.69	0.85	3.71 ± 0.73	0.88	3.23 ± 0.98
	natural ^b,n,^***	3.95 ± 0.77	0.89	3.47 ± 0.80	0.88	3.64 ± 0.80	0.89	3.68 ± 0.81
	*M* ± *SD_d_*	2.97 ± 1.09	a,l ***	3.41 ± 0.80	l,b ***	3.51 ± 0.85	a,b ***	
t	d	nr	cr	c*d	c*nr	d*nr	c*cr	d*cr
p|ɳ^2^ F|df								
***|0.029 424,81|2	***|0.012 183.34|2	***|0.001 20.57|1	***|0.007 193.95|1	***|0.017 124.15|4	***|0.004 66.73|2	0.40|<0.001 0.91|2	***|0.009 130.36|2	0.78|<0.001 0.26|2
		*M* ± *SD*	*α*	*M* ± *SD*	*α*	*M* ± *SD*	*α*	*M* ± *SD_c_*
stimulating	built ^b,m,^***	2.53 ± 0.91	0.90	2.76 ± 0.76	0.93	2.57 ± 0.84	0.92	2.62 ± 0.84
	mixed ^m,n,^***	2.33 ± 0.80	0.93	3.73 ± 0.67	0.90	3.58 ± 0.76	0.92	3.21 ± 0.97
	natural ^b,n,^***	4.16 ± 0.64	0.95	3.47 ± 0.82	0.91	3.91 ± 0.73	0.93	3.85 ± 0.79
	*M* ± *SD_d_*	3.01 ± 1.14	a,l ***	3.32 ± 0.85	l,b	3.36 ± 0.96	a,b ***	
t	d	nr	cr	c*d	c*nr	d*nr	c*cr	d*cr
p|ɳ^2^ F|df								
***|0.071 1335,40|2	***|0.005 98.19|2	0.10|<0.001 2.61|1	***|0.006 224.85|1	***|0.029 255.57|4	***|0.005 84.12|2	0.20|<0.001 1.63|2	***|0.007 136.00|2	*|<0.001 4.08|2
		*M* ± *SD*	*α*	*M* ± *SD*	*α*	*M* ± *SD*	*α*	*M* ± *SD_c_*
activating	built ^b,m,^**	3.01 ± 0.88	0.74	3.01 ± 0.88	0.84	3.45 ± 0.66	0.79	3.27 ± 0.82
	mixed ^m,n,^***	2.70 ± 0.78	0.86	3.35 ± 0.66	0.79	3.57 ± 0.68	0.85	3.20 ± 0.80
	natural ^b,n,^***	3.35 ± 0.70	0.89	3.10 ± 0.79	0.86	3.12 ± 0.77	0.84	3.19 ± 0.76
	*M* ± *SD_d_*	3.02 ± 0.83	a,l ***	3.27 ± 0.74	l,b	3.38 ± 0.76	a,b ***	
t	d	nr	cr	c*d	c*nr	d*nr	c*cr	d*cr
p|ɳ^2^ F|df								
***|0.003 40,14|2	***|0.009 110.34|2	***|<0.001 3.97|1	***|0.004 93.19|1	***|0.013 74.99|4	***|0.013 151.80|2	***|0.001 7.71|2	***|0.012 140.55|2	*|<0.001 3.96|2
		*M* ± *SD*	*α*	*M* ± *SD*	*α*	*M* ± *SD*	*α*	*M* ± *SD_c_*
preference	built ^b,m,^***	2.84 ± 1.07		3.02 ± 0.95		2.88 ± 1.01		2.91 ± 1.02
	mixed ^m,n,^***	2.61 ± 0.98		4.04 ± 0.77		3.92 ± 0.91		3.52 ± 1.10
	natural ^b,n,^***	4.45 ± 0.71		3.74 ± 0.91		4.20 ± 0.80		4.31 ± 0.86
	*M* ± *SD_d_*	3.30 ± 1.24	a.l ***	3.60 ± 0.98	l,b	3.67 ± 1.07	a,b ***	
t	d	nr	cr	c*d	c*nr	d*nr	c*cr	d*cr
p|ɳ^2^ F|df								
***|0.063 1067.16|2	***|0.004 62.90|2	0.83|<0.001 0.05|1	***|0.011 361.67|1	***|0.027 216.03|4	***|0.004 37.37|2	0.80|<0.001 0.072|2	***|0.007 115.78|2	0.80|<0.001 0.23|2
		*M* ± *SD*	*α*	*M* ± *SD*	*α*	*M* ± *SD*	*α*	*M* ± *SD_c_*
mental fatigue	built ^b,m,^***	2.41 ± 1.18		2.29 ± 1.08		2.70 ± 1.23		2.47 ± 1.20
	mixed ^m,n^	2.29 ± 1.10		1.98 ± 1.03		2.09 ± 1.03		2.12 ± 1.06
	natural ^b,n,^***	1.84 ± 1.16		2.09 ± 1.10		1.92 ± 1.13		1.95 ± 1.13
	*M* ± *SD_d_*	2.18 ± 1.17	a,l	2.12 ± 1.08	l,b	2.24 ± 1.20	a,b	
t	d	nr	cr	c × d	c × nr	d × nr	c × cr	d × cr
p|ɳ^2^ F|df								
***|0.003 37.77|2	0.22|<0.001 1.53|2	***|0.002 50.16|1	***|0.001 21.74|1	***|0.005 27.77|4	***|0.001 13.28|2	***|<0.001 4.99|2	***|0.002 16.744|2	***|0.001 10.85|2

Note. t = types; d = detailed; nr = nature relatedness; cr = city relatedness; b,m = contrast built × mixed; m,n = contrast built × nature; b,n = contrast mixed × nature; a,l = contrast average × livability; l,b = contrast livability × bird’s-eye view, a,b = contrast average × bird’s-eye view; *** < 0.001; ** < 0.01; * < 0.05; n.s. = not significant.

## Data Availability

The data presented in this study are openly available as Supplemental Material.

## Supplementary Materials

The following supporting information can be downloaded at: https://www.mdpi.com/article/10.3390/ijerph19137606/s1. Figure S1. Photographs of the Places; Figure S2. Graphical Illustrations of Interaction Effects; Table S1. Classification of the Places considered; Table S2. Preferences of Places in %; Table S3. Descriptive Statistics, *t*-test, Correlations, and Cronbach’s α for Nature and City Relatedness, Experience and Self; Table S4. Nature and City Relatedness Scale; Table S5. Exploratory Factor Analyses for Stimulating and Activating Effects of Each Place; Table S6. Fit Criteria for the Nature and City Relatedness Scales; Table S7. Correlations Between Perceived Restorativeness with Stimulating and Activating Effects, Preference and Mental Fatigue; Table S8. Participants Addressed Cues of the Places.
