# Supplementary material for: Shedding Light onto the City Blues Myth—The Potential of Stimulating and Activating Effects of Urban Public Spaces and the Role of City Relatedness
Source: Int J Environ Res Public Health. 2022 Jun 21;19(13):7606. doi: 10.3390/ijerph19137606 (PMC9266095; doi:10.3390/ijerph19137606)
Supplement: Supplementary file 1 [file ijerph-19-07606-s001.zip › ijerph-1759975-supplementary/supplemental material/figures.pdf]

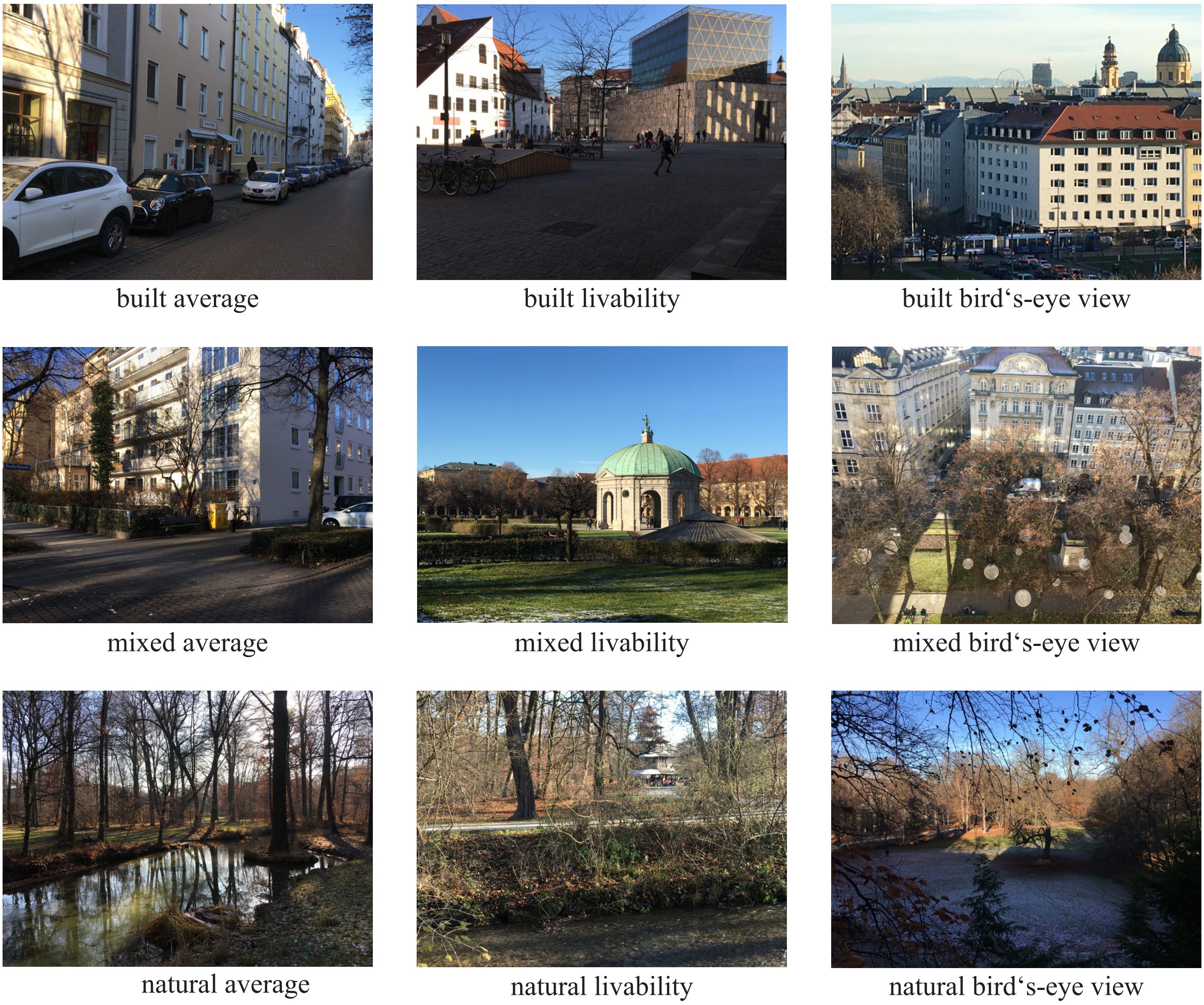

Figure S1. Photographs of the Places

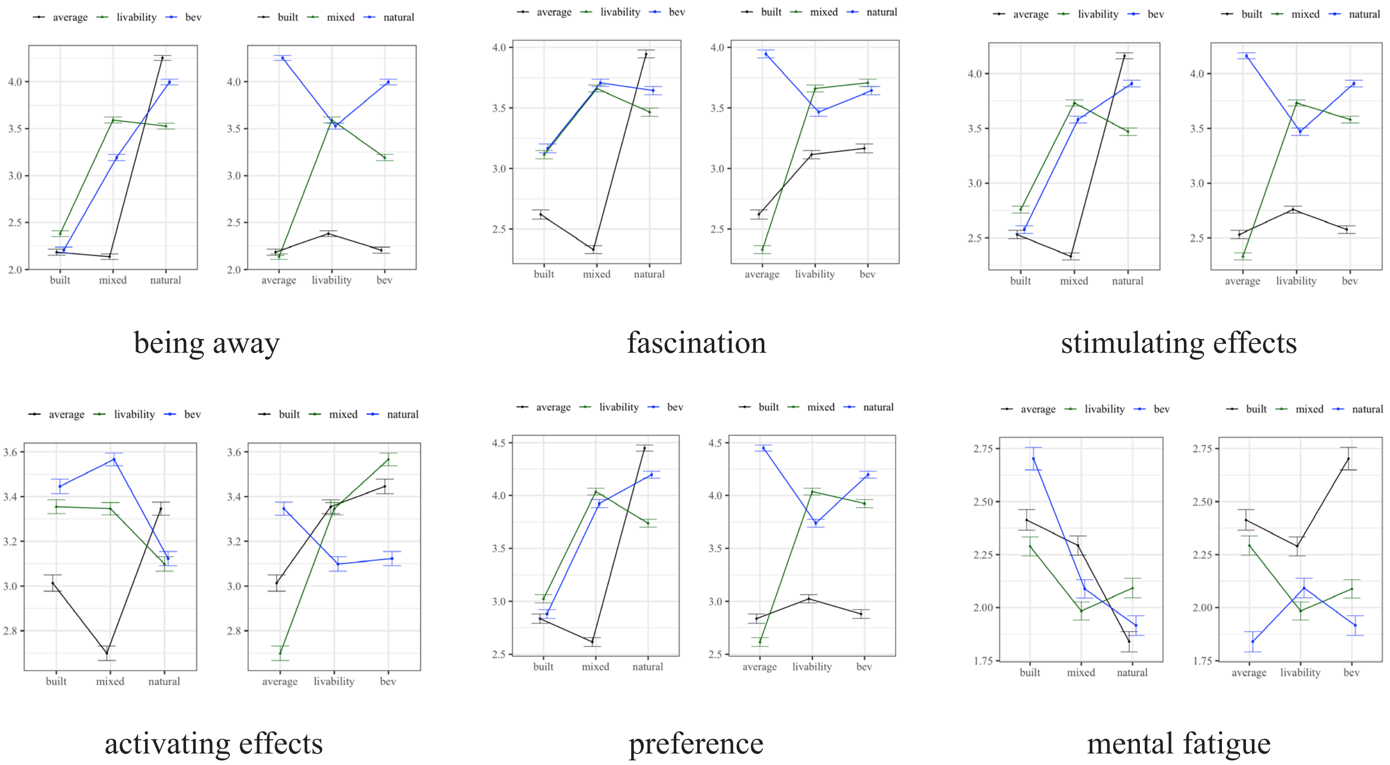

Figure S2. Graphical Illustrations of Interaction Effects.
