# Supplementary material for: Shedding Light onto the City Blues Myth—The Potential of Stimulating and Activating Effects of Urban Public Spaces and the Role of City Relatedness
Source: Int J Environ Res Public Health. 2022 Jun 21;19(13):7606. doi: 10.3390/ijerph19137606 (PMC9266095; doi:10.3390/ijerph19137606)
Supplement: Supplementary file 1 [file ijerph-19-07606-s001.zip › ijerph-1759975-supplementary/supplemental material/tables.pdf]

Supplement

Table S1

Classification of the Places considered

livability|PSD	cues
nature	nature quality[ma, na, ml, nl, mb, nb]
	wild[na, nl, nb]
	free growing lawns[na, nl]
	pond, canal[na, nl]
	hilly[nb]
	wooded pasture quality[na, nl, nb]
	path made of gravel[ml, nl, nb]
	lots of trees[ma, na, ml, nl, mb, nb]
	many bushes[ma, na, ml, nl]
	city park characteristic[na, ml, nl, mb, nb]
	plane, well-cut grass[na, ml, mb, nb]
	cut lawns[na, nb]
culture	fountains[bl, ml]
	sculptures[bl, ml, nl, mb]
	exhibitions[mb]
	historical buildings[ba, ma, bl, ml, bb, mb]
prospect	wide view[ba, ml, bb, nb]
	vistas[na, ml, bb, mb]
	different perspectives[ma, bl]
social	entertainment[ba, bl]
	restaurants|café[ba]
	seats & benches[ba, ma, bl, ml, nl, mb]
	tables[ba, nl]
	plenty of people[bl, ml, nl, bb, mb]
	places where people can gather[bl, ml, nl, mb]
	watching people[ba, bl, ml, mb, nl]
space	spacious[bl, ml, mb, nb]
	areas not crossed by paths[ba, na, bl]
	sunny places[ba, ma, na, bl, ml, nl, bb, mb, nb]
	shady places[ba, ma, na, bl, ml, nl, bb, mb, nb]
rich in information	lot of different elements[ma, bl, bb, mb]
	detailed information[ba, ma, bl, ml, mb, nl, bb]
	no repetitions[bl, ml, nl, bb]
refuge	effortless information[ba, na, nb]
	attractive information[na, bl, ml, bb, mb, nb]
	unusual information[bl, ml, mb, nl, bb]
serene	silent & calm[na, nb]
	no bikes[na, ml]
	no mopeds[na, bl, ml, nl, nb]
	no car[na, bl, ml, nl, nb]
	no traffic noise[na, bl, ml, nl, nb]
	clean[na, bl, ml, nl, bb, mb, nb]
	well maintained[ba, ma, na, bl, ml, bb, mb, nb]
	feels safe[ba, ma, na, bl, ml, nl, mb, nb]

Note. PSD = perceived sensory dimension; [ba] = built-average; [ma] = mixed-average; [na] = natural-average; [bl] = built-livability; [ml] = mixed-livability; [nl] = natural-livability; [bb] = built-bird's-eye view; [mb] = mixed-bird's-eye view; [nb] = natural-bird's-eye view.

Table S2

Preferences of Places in %

	average			livability			bird's-eye view		
	1	2	3	1	2	3	1	2	3
built	11,24	38,15	50,60	12,04	24,10	63,86	6,02	13,25	84,34
mixed	04,01	49,80	46,18	59,44	33,33	07,23	46,59	49,80	03,61
natural	84,74	12,05	3,21	28,51	42,57	28,92	47,39	36,95	15,66

Note. 1 = first preference; 2 = second preference; 3 = third preference

Table S3

Descriptive Statistics, t-Test, Correlations, and Cronbach's α for Nature and City Relatedness, Experience and Self

relatedness				experience				self			
M±SD		p	d	*M±SD*		p	d	*M±SD*		p	d
nature	city			nature	city			nature	city		
3,46±0,59	3,13±0,57	<.001	.57	3,34±0,68	3,38±0,60	.50	.06	3,55±0,64	2,94±0,66	<.001	.59
α		r		*α*		r		*α*		r	
.85	.84	-.02		.69	.66	-.06		.82	.80	.09	

Note. Reference means for the NRS range from 3.28 to 3.40 (SD 0,58 – 0.68) and from 3.00 to 3.56 (SD 0.85 – 1.01) for the short form version of the NRS [46]. The mean of nature relatedness in the present study therefore lies between the ranges for the short form version, but outside the range of the original scale. However, the z-value reveals no significant deviation (z < 1.96) from its highest mean (3.40).

Table S4

Nature and City Relatedness Scale

Nature Relatedness Scale	City Relatedness Scale
I enjoy being outdoors, even in unpleasant weather.	I like to move in the city, even in unpleasant weather.
My ideal vacation spot would be a remote, wilderness area.	My ideal vacation spot would be a vibrant metropolis.
I always think about how my actions affect the environment.	I think about how to improve city life.
I enjoy digging in the earth and getting dirt on my hands.	I like to make use of the diverse offers of the city.
My connection to nature and the environment is part of my spirituality.	My connection to the city and the city life is part of my spirituality.
I am very aware of environmental issues.	I am very aware of city affairs.
I take notice of wildlife wherever I am.	I take notice of cityscape wherever I am.
I don't often go out in nature.	I don't often go out in the city.
I am not separate from nature, but part of nature.	I am not separate from the city, but part of the city.
The thought of being deep in the woods, away from civilization, is frightening.	The thought of being in the busy, dense center of the city is frightening.
My feelings about nature do not affect how I live my life.	My feelings about the city do not affect how I live my life.
Even in the middle of the city, I notice nature around me.	Even in the middle of nature, I think about the amenities in the city.
My relationship to nature is an important part of who I am.	My relationship to the city is an important part of who I am.
I feel very connected to all living things and the earth.	I feel very connected to the city.

Note. The facet 'perspective' was not considered.

Table S5

Exploratory Factor Analyses for Stimulating and Activating Effects of Each Place

	average		livability		bird's-eye view		
	B|KMO	MAP|H|E	B|KMO	MAP|H|E	B|KMO	MAP|H|E	
built	***|.94	2|2|2	***|.92	2|2|2	***|.92	2|2|2	
mixed	***|.92	2|2|2	***|.91	2|3|2	***|.92	2|2|2	
natural	***|.88	2|3|2	***|.92	2|2|2	***|.91	2|3|2	
	s	a	s	a	s	a	item
built	.94		.79		.83		1
	.74		.58		.62		2
	.77		.57		.60		3
	.82		.85		.85		4
	.74		.63		.68		5
	.93		.90		.89		6
	.91		.87		.96		7
	.57		.48		.50		8
		.81		.74		.68	9
		.55		.61		.89	10
		.86		.83		.96	11
		.86		.80		.50	12
		.50		.64		.40	13
mixed	.84		.95		.86		1
	.58		.68		.61		2
	.69		.68		.68		3
	.87		.73		.84		4
	.71		.54		.70		5
	.96		.86		.96		6
	.97		.88		.91		7
	.49		.31	.41	.36	.40	8
		.75		.76		.63	9
		.57		.40	.42	.42	10
		.80		.85		.86	11
		.87		.59		.85	12
		.61		.40		.58	13
natural	.86		.91		.95		1
	.63		.69		.72		2
	.65		.61		.70		3
	.76		.83		.78		4
	.65		.69		.57		5
	.90		.98		.96		6
	.90		.95		.96		7
	.43		.62		.34		8
		.76		.82		.75	9

	.54		.63		.53	10
	.74		.83		.72	11
	.62		.67		.70	12
.56		.48	.33	.63	.21	13

stimulating effects

1 This place creates a pleasant atmosphere.
2 This place has stimulating characteristics.
3 This place has a stimulating appearance.
4 This place invites relaxation.
5 In this place I recognize myself.
6 This place regulates my emotions in a positive sense.
7 This place regulates my thoughts in a positive sense.
8 I would meet my friends at this place, so that they can learn more about me.

activating effects

9 This place encourages social life.
10 This place provides amenities.
11 At this place you are at the cutting edge.
12 This place provides a broad scope for action.
13 This place has something invigorating.

Note. bird = bird's-eye view; B = Bartlett-test; H = Horn's parallel analysis; E = eigenvalue; s = factor loadings stimulating effects; a = factor loadings activating effects.

Table S6

Fit Criteria for the Nature and City Relatedness Scales

	χ^2	df	p	CFI	RMSEA|CI	SRMR
nature relatedness	199,45	76	<.001	.881	.081|.067-.095	.061
nature relatedness*	161,13	75	<.001	.917	.068|.053-.082	.054
city relatedness	156,33	76	<.001	.917	.065|.051-.080	.053

Note. * = items 'I always think about how my actions affect the environment.' and 'I am very aware of environmental issues.' were correlated.

Table S7

Correlations Between Perceived Restorativeness with Stimulating and Activating Effects, Preference and Mental Fatigue

	being away			
	stimulating effects	activating effects	preference	mental fatigue
built average	.80***	.56***	.67***	.10
mixed average	.81***	.55***	.66***	.04
natural average	.80***	.40***	.69***	-.17*
built livability	.71***	.34***	.60***	.07
mixed livability	.74***	.40***	.65***	-.04
natural livability	.80***	.49***	.73***	-.08
built bird	.72***	.34***	.62***	-.12
mixed bird	.73***	.40***	.57***	.00
natural bird	.82***	.49***	.75***	-.04
	fascination			
	stimulating effects	activating effects	preference	mental fatigue
built average	.82***	.70***	.74***	.09
mixed average	.78***	.69***	.69***	.04
natural average	.80***	.60***	.61***	.01
built livability	.73***	.61***	.75***	-.02
mixed livability	.73***	.55***	.63***	-.05
natural livability	.78***	.60***	.67***	.04
built bird	.77***	.69***	.74***	.02
mixed bird	.77***	.68***	.69***	-.11
natural bird	.80***	.70***	.69***	.03

Note. bird = bird's-eye view; ***<.001; *<.05.

Table S8

Participants Addressed Cues of the Places

	average^{n}	livability^{n}	bird's-eye view^{n}
built	cars^{22}	people^{9}	(lot of) traffic^{28}
	(unanimated) (narrow)	(lot of) concrete^{8}	tramway^{18}
	(dense) street^{8}	(tall) paved place^{5}	(lot of) cars^{15}
	block houses^{8}	children (let me think of	traffic jam^{10}
	traffic^{3}	my work)^{3}	noise^{8}
	concrete^{3}	(lot of) block of houses^{3}	block of houses^{6}
	not enough parking places^{3}	bicycles^{3}	(lot of) people^{6}
	lot of people live there^{2}	cold (facades)^{2}	tall buildings^{4}
	dark^{2}	tall white façade^{2}	buildings in the foreground^{4}
	hectic^{2}	contrast new vs. old^{1}	concrete^{4}
	unattractive (old)	straight lines^{1}	ferris wheel^{4}
	architecture^{2}	sun^{1}	cranes^{3}
	tall buildings^{2}	buildings^{1}	hectic^{3}
	café^{1}	noise^{1}	unattractive architecture^{3}
	straight street^{1}	no nature^{1}	density^{2}
	tree^{1}	geometric shapes^{1}	dull^{1}
	attractive street scene^{1}	tall box-shaped building^{1}	barren^{1}
	no nature^{1}	wall on the right side^{1}	traffic light^{1}
	showcase^{1}	advertising poster^{1}	lanterns^{1}
	no privacy^{1}	darkness^{1}	undecorated^{1}
	lot of action^{1}		smell^{1}
	asphalt^{1}		lot of movement^{1}
	shops^{1}		
mixed	block of houses^{7}	architectural style^{4}	(nearby) cars, traffic (noise)^{14}
	letterbox^{7}	green field^{1}	(tall), (massive), (beautiful)
	cars^{7}	roof of all^{1} buildings^{1}	facades|buildings^{7}
	(tall) buildings^{6}	people^{1}	parc in the city^{2}
	(broad) streets^{5}	bench^{1}	dense of row houses^{2}
	housing conditions^{4}	cold (snow) ^{1}	people^{2}
	(small) balconies^{3}	aesthetics^{1}	lanterns on the tree^{1}
	corner house^{2}		monuments^{1}
	bench^{2}		leaves^{1}
	graffities^{1}		the small parc somewhat leaves a
	artificial green^{1}		melancholic impression^{1}
	unattractive architecture^{1}		sun^{1}
	dull, old building^{1}		
	oppressive^{1}		
	little light^{1}		
	plants^{1}		
	chaotic^{1}		
natural	water^{4}	crowd of people^{8}	trees^{3}
	trees^{2}	pavilion^{8}	shadow^{2}
	nature (experience)^{2}	concrete pathway^{7}	bare trees^{2}
	forest	chaotic^{3}	sun
	shadow	not inviting^{1}	green
	fresh air	ripples of water^{1}	people
		bare tress^{1}	silence
			gravelly soil

	fresh air<br>calm atmosphere

Note. n = numbers of time mentioned.
